# Effects of EEG neurofeedback and training interventions on golf putting performance: a systematic review and meta-analysis

**DOI:** 10.3389/fpsyg.2026.1736851

**Published:** 2026-04-23

**Authors:** Zhenjun Li, Heng Liu, Chao Liu, Fuyi Liu, Baohua Liu

**Affiliations:** 1 School of Tourism and Public Administration, Zhuhai College of Science and Technology, Zhuhai, China; 2 School of Sports Training, Tianjin University of Sport, Tianjin, China; 3 School of Sport, Exercise and Health Sciences, Loughborough University, Leicestershire, United Kingdom; 4 School of Social Sports, Tianjin University of Sport, Tianjin, China

**Keywords:** electroencephalography, golf, meta-analysis, neurofeedback, putting performance

## Abstract

**Background:**

Electroencephalography neurofeedback (EEG-NFT) is used to modulate brain function to enhance golf putting performance. However, the effectiveness of EEG-NFT and its relationship with non-EEG training interventions in improving putting performance remain unclear.

**Methods:**

Following the Preferred Reporting Items for Systematic Reviews and Meta-Analyses (PRISMA) guidelines and the International Prospective Register of Systematic Reviews (PROSPERO) registration (CRD420251068586), this study systematically searched multiple databases up to 8 June 2025. Randomized controlled trials (RCTs) investigating EEG-NFT and other training interventions for golf putting were included. The meta-analyses employed random-effects models, with subgroup analyses conducted to examine moderators.

**Results:**

A total of 12 RCTs involving 424 participants were included. The overall analysis revealed a significant positive effect on putting performance (standardized mean difference, *SMD* = 1.33; 95% CI: 0.36–2.29; *p* = 0.007), though with substantial heterogeneity (*I^2^* = 94%). Subgroup analyses demonstrated that skill level significantly moderated the intervention effects (*p* = 0.0003), with novices showing the largest improvement (*SMD* = 2.10). Crucially, intervention efficacy was strongly dependent on both the anatomical specificity and frequency band targeting: central region-sensorimotor rhythm (Cz-SMR)-NFT demonstrated robust positive effects (*SMD* = 1.12, 95% CI: 0.59–1.66), while inhibitory protocols targeting Mu rhythms (*SMD* = −1.84) and frontal midline theta (*SMD* = −1.92) showed significant negative effects. Non-EEG training interventions also exhibited positive effects on putting performance, with skill practice-based interventions showing comparable efficacy to effective EEG-NFT protocols in novice golfers.

**Conclusion:**

EEG-NFT can enhance putting performance, but success requires precise protocol selection aligned with skill level and neural targets. Cz-SMR enhancement appears to be reliable, whereas non-specific suppression may be detrimental. Future research should establish standardized protocols and conduct larger RCTs to validate these findings.

**Systematic review registration:**

https://www.crd.york.ac.uk/prospero/display_record.php?RecordID=1068586, identifier (CRD420251068586).

## 1 Introduction

There is a slang saying in golf: *driver for show, putter for money* (Jiménez-Ortega et al., 2020; Brouillette, 2010). Although a powerful drive captures spectators’ attention, the putter is the instrument that determines victory. Putting accounts for about 43% of the total strokes in a round (Tanaka and Iwami, 2018; Richardson et al., 2015), and with an accurate putting technique, getting the ball into the hole in fewer strokes may be an effective way to improve performance (Shim et al., 2019). Unlike a golf swing that relies heavily on physical power and swing mechanics, putting is a complex interplay of technical precision, psychological resilience, neurocognitive control, and environmental adaptation (McHugh et al., 2024; Chen et al., 2022). At the technical level, parameters such as backswing amplitude standardization and impact point accuracy directly shape the ball’s rolling trajectory (Yang et al., 2024; Liu et al., 2025). At the psychological level, the ability to regulate anxiety under pressure dictates movement stability (Cooke et al., 2014); at the neurocognitive level, the duration of the modulation of SMR forms the neural foundation of consistent performance; and at the greenside reading level, perceptual biases toward green speed or slope can introduce strategic errors (Wu et al., 2024b; Wu et al., 2024a). Complicating matters further, the dynamic complexity of the green environment—where grass patterns, slopes, and moisture interact to alter ball roll—makes purely technical or environmental quantification of putting performance impractical (Stephens et al., 2021; Hasegawa et al., 2024). This multidimensional challenge has shifted research focus toward neurocognitive mechanisms, with electroencephalography (EEG) emerging as a pivotal tool for unraveling the role of the brain in optimal putting, owing to its operationalizability and mechanistic clarity (Wang et al., 2023; Ji et al., 2019; Afrash et al., 2023).

EEG provides a non-invasive window into the neural dynamics underlying motor performance. The technique captures synchronized electrical activity from cortical neural networks, with specific frequency bands reflecting distinct cognitive and motor processes (Cai et al., 2024). EEG activity includes neural oscillations measured using EEG, characterized by frequency bands (such as alpha, beta, theta, sensorimotor rhythm [SMR], and Mu) and spatial distribution. In golf putting, expertise is characterized by distinctive EEG signatures. Skilled golfers demonstrate enhanced SMR (12–15 Hz) synchronization and optimized alpha-wave modulation (8–12 Hz) during movement preparation, particularly over the central and frontal regions (Lu et al., 2025). This efficient neural profile contrasts with the disorganized cortical activity observed in less skilled players (Wright et al., 2013). Furthermore, under competitive pressure, elite golfers maintain stable EEG patterns while amateurs exhibit elevated prefrontal midline theta (FMT, 4–7 Hz) power–a marker of cognitive overload that compromises performance stability (Wang et al., 2020). During high-stakes moments, amateur golfers show a marked elevation in FMT power, a marker of increased cognitive effort and anxiety that disrupts automatic movement (Chen et al., 2022). Elite golfers, however, maintain relatively stable EEG patterns: their SMR remains consistent, and FMT elevation is minimized, allowing them to preserve motor efficiency despite stress (Afrash et al., 2023). This contrast highlights the potential of EEG to identify “neural resilience” markers that distinguish elite from sub-elite performance. Neural resilience is defined as the capacity to maintain stable, task-optimal oscillatory patterns under conditions of psychological pressure or cognitive demand. In golf putting, neural resilience is empirically characterized by two key EEG signatures: (1) the stability of SMR synchronization, which underpins consistent motor execution, and (2) the effective regulation of frontal midline theta power, which minimizes performance-disrupting cognitive interference. EEG-NFT: an intervention in which individuals receive real-time feedback on specific EEG frequency bands to learn self-regulation of cortical activity. Parallel to EEG-NFT, training interventions have long been the mainstream practical strategies for enhancing golf putting performance, encompassing skill practice-based approaches and cognitive control training (Abdoli et al., 2025; Gallicchio and Ring, 2019; Wang et al., 2024; Zhu et al., 2011). These non-EEG interventions (training approaches that do not involve neurofeedback but may include EEG measurement for mechanistic insight) focus on optimizing technical proficiency, motor memory, and cognitive regulation through targeted practice or psychological modulation, complementing the neurocognitive targeting of EEG-NFT. Despite their widespread application, the efficacy of non-EEG training interventions, and their comparative effectiveness relative to EEG-NFT, remain insufficiently synthesized in the existing literature.

Mu rhythm (8–13 Hz) is an oscillatory pattern recorded over the sensorimotor cortex that is suppressed during the execution, observation, or imagination of motor actions (Sabate et al., 2012). Previous studies have demonstrated that Mu rhythm desynchronization (ERD) correlates with the preparation and execution of voluntary movements, while synchronization (ERS) is associated with movement inhibition and resting states (Debnath et al., 2019). Research has found that athletes with higher motor skill proficiency exhibit more pronounced ERD during motor execution, indicating that the Mu rhythm reflects the activation efficiency of the motor cortex (Filho et al., 2021). Existing studies show inconsistent findings, with some reporting significant improvements following SMR enhancement, while others show null or even detrimental effects from protocols targeting Mu or frontal theta rhythms (Van Noordt et al., 2016). This heterogeneity likely stems from a failure to systematically account for critical moderating variables, particularly athlete expertise, anatomical specificity of intervention targets, and training dosage. Moreover, the field lacks a comprehensive quantitative synthesis to determine overall effect sizes and elucidate the precise conditions under which EEG neurofeedback training (NFT) proves effective.

This systematic review and meta-analysis aim to quantitatively synthesize evidence on EEG activity in golf putting performance. The study will quantify associations between key EEG metrics and putting outcomes, examine skill level as a moderator, evaluate EEG neurofeedback efficacy, and identify methodological sources of heterogeneity. The findings will clarify neurocognitive mechanisms of putting performance and support the development of targeted training protocols based on individual neural profiles and skill levels.

This systematic review and meta-analysis aims to quantitatively synthesize evidence on EEG activity in golf putting performance, with objectives: (1) pooled effect estimation to quantify associations between key EEG metrics and the putting outcomes; (2) moderator analysis to examine skill level as a moderator; (3) differential EEG-NFT protocol efficacy to evaluate the effectiveness of EEG-NFT and other training interventions; and (4) heterogeneity source identification to clarify methodological variations. The findings will elucidate neurocognitive mechanisms underlying putting performance and support the development of targeted training protocols based on individual neural profiles and skill levels.

## 2 Materials and methods

The study protocol for this systematic review and meta-analysis was prospectively registered in the International Prospective Register of Systematic Reviews (PROSPERO) under the registration number CRD420251068586. The review was conducted in strict accordance with the guidelines of Preferred Reporting Items for Systematic Reviews and Meta-Analyses (PRISMA) to ensure transparency and methodological rigor in study selection, data extraction, and quantitative synthesis.

### 2.1 Inclusion and exclusion criteria

#### 2.1.1 Inclusion criteria

1. Population (P): Studies involving golfers (amateur or professional) without neurological or motor impairments affecting putting.
2. Intervention (I): Studies evaluating training interventions for golf putting performance, including (a) EEG neurofeedback interventions (real-time feedback based on EEG signals) and (b) non-EEG training interventions (skill practice and cognitive control training) that include EEG measurement.
3. Comparison (C): Studies comparing EEG measures between skill levels or performance outcomes, or evaluating EEG interventions.
4. Outcomes (O): Quantitative measures of putting performance and corresponding EEG data.
5. Study Design (S): This systematic review included only Randomized Controlled Trials (RCTs) that evaluated either (a) EEG-NFT or (b) non-EEG training interventions for golf putting performance. This study excluded observational designs (cohort studies and case–control designs), qualitative studies, case reports, narrative reviews, systematic reviews, and meta-analyses to prioritize primary research evidence with the highest methodological rigor for evaluating intervention efficacy.

#### 2.1.2 Exclusion criteria

1. Studies not focused on golf putting.
2. Studies lacking quantitative EEG data or performance metrics.
3. Non-English full-text articles (after screening titles/abstracts).
4. Case reports and narrative reviews.

### 2.2 Literature search strategy

#### 2.2.1 Identify databases

Multiple relevant databases were selected to ensure a comprehensive and rigorous search. The databases included in the search were PubMed, Web of Science, Scopus, and EBSCO.

#### 2.2.2 Search terms

To conduct a thorough and systematic review, this study performed a comprehensive literature search utilizing Medical Subject Headings (MeSH) and keywords structured around the Population, Intervention, Comparison, and Outcome (PICO) framework. To ensure complete transparency and reproducibility, the detailed search strategy, including specific search terms, databases utilized, and search strings, is documented in Supplementary file 1.

#### 2.2.3 Combined search form (using the Scopus database as examples)

In Scopus, the search terms were: TITLE-ABS-KEY (golf) OR TITLE-ABS-KEY (golf AND putting) OR TITLE-ABS-KEY (golf AND putt) OR TITLE-ABS-KEY (golf AND putting AND performance) OR TITLE-ABS-KEY (golf AND putting AND accuracy) OR TITLE-ABS-KEY (golf AND putting AND success) AND (TITLE-ABS-KEY (electro-encephalography) OR TITLE-ABS-KEY (EEG) OR TITLE-ABS-KEY (brain AND wave) OR TITLE-ABS-KEY (alpha AND wave) OR TITLE-ABS-KEY (beta AND wave) OR TITLE-ABS-KEY (theta AND wave) OR TITLE-ABS-KEY (neural AND oscillation) OR TITLE-ABS-KEY (neurofeedback) OR TITLE-ABS-KEY (EEG AND feedback)).

#### 2.2.4 Scope of search

Studies published through 8 June 2025 were systematically retrieved, encompassing both journal articles and dissertations. To mitigate publication bias and ensure comprehensiveness, secondary searches were performed on the bibliographies of included studies and relevant review articles.

### 2.3 Study selection and data extraction

#### 2.3.1 Literature screening

**Figure 1:**
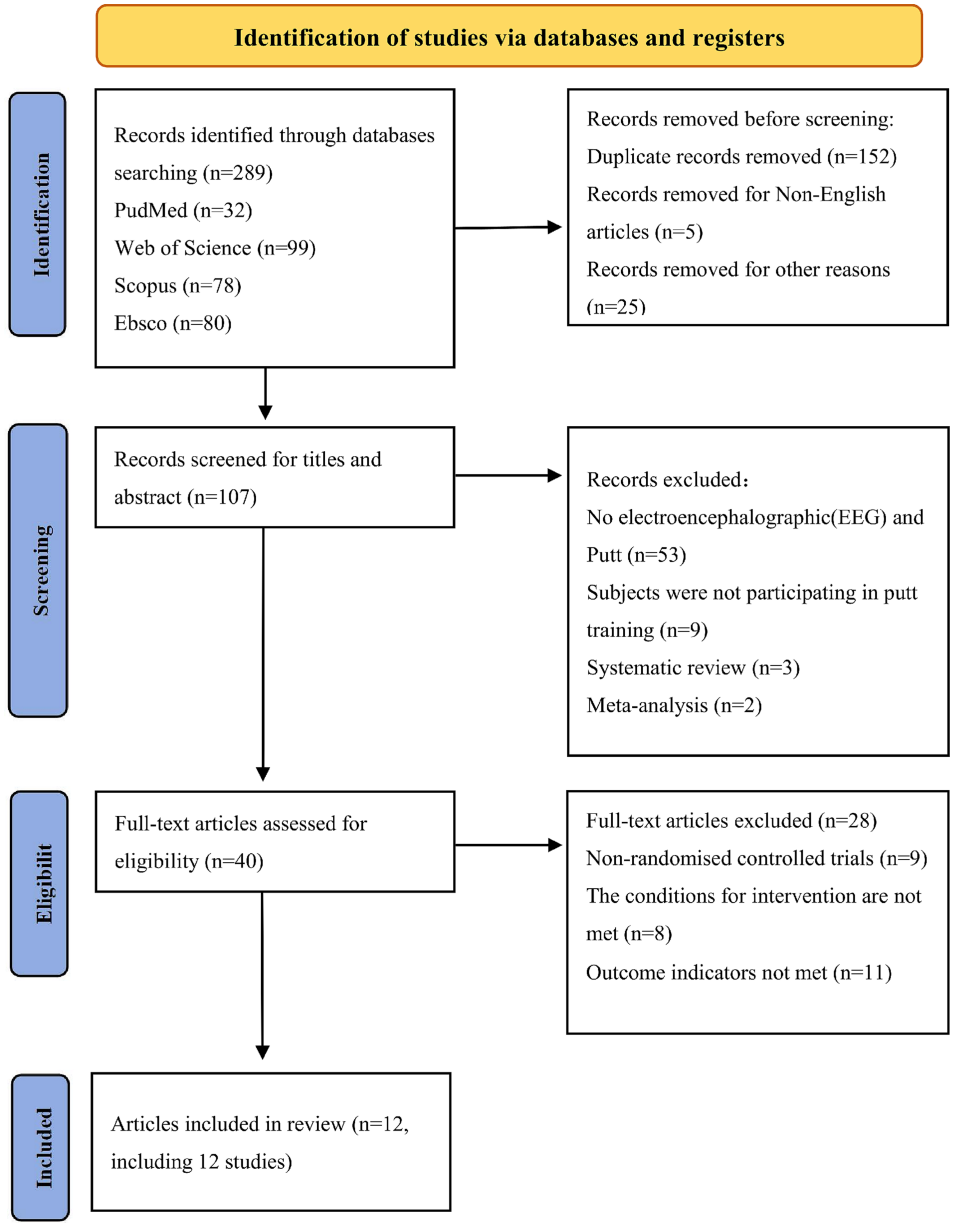
Literature screening flowchart.

Title and Abstract Screening: Two reviewers (Z.L. and H.L.) independently screened the titles and abstracts of all retrieved records against the predefined inclusion criteria. Each reviewer documented their decisions, and disagreements were resolved through discussion. Full-Text Assessment: Two reviewers (C.L. and F.L.) independently assessed the full texts of potentially eligible studies against the inclusion criteria. Reasons for exclusion at this stage were documented for each study. Disagreement Resolution: If the two reviewers at any stage could not reach consensus, the disagreement was referred to a senior reviewer (B.L.) for final decision. Verification: A third reviewer (C.L.) verified the final list of included studies and cross-checked exclusion reasons against the PRISMA guidelines. The entire screening process was managed using EndNote X9 (Thomson Reuters, Carlsbad, CA, USA) for duplicate removal and organization. A PRISMA flow diagram (Figure 1) documents the number of studies at each stage and provides reasons for exclusion.

#### 2.3.2 Literature extraction

Data extraction was performed independently by two reviewers (Z.L. and C.L.) using a standardized data extraction form that was piloted on three included studies. The form captured the following information: (1) study characteristics (author, year, country, and study design), (2) participant characteristics (sample size, age, skill level, and handicap), (3) intervention details (type, protocol, duration, and control condition), (4) EEG measures (recording sites, frequency bands, power metrics, and processing methods), (5) putting performance outcomes (metrics and measurement conditions), and (6) statistical data (means, standard deviations, sample sizes, and effect sizes). After independent extraction, the two reviewers cross-checked all the extracted data for accuracy. Discrepancies were resolved through discussion, and a third reviewer (B.L.) was consulted when necessary. Data were compiled using Microsoft Excel 2021 (Microsoft Corporation, Redmond, WA, USA).

### 2.4 Assessment of literature quality

Risk of bias was assessed independently by two reviewers (Z.L. and H.L.) using the Cochrane Collaboration’s risk of bias tool (RoB 2). Each reviewer evaluated the included studies across seven domains, namely: (1) random sequence generation, (2) allocation concealment, (3) blinding of participants and personnel, (4) blinding of outcome assessment, (5) incomplete outcome data, (6) selective reporting, and (7) other sources of bias. At all stages of screening and data extraction, disagreements between the reviewers were resolved through discussion. Reviewers first attempted to reach consensus by re-examining the original study and discussing the rationale for their judgments. When consensus could not be achieved, a third reviewer (B.L.) made the final decision. This process ensured that all included studies and extracted data met the agreed-upon criteria.

### 2.5 Statistical analysis

Statistical analyses were conducted using Review Manager 5.4 (The Cochrane Collaboration, Oxford, UK). For continuous outcomes, the standardized mean difference (SMD) was calculated, with corresponding 95% confidence intervals (95% CIs) extracted for each outcome. To integrate findings from studies employing different measurement scales (success rate, radial error, and EEG power values), this research adopted standardized mean difference as a unified effect size metric. All putting performance and EEG outcome measures were categorized as positive and negative indicators. Positive indicators: Higher values represent better performance and more beneficial neuromodulation (putting success rate, number of successful putts, enhanced sensorimotor rhythm power in target brain regions). Negative indicators: Lower values represent better performance and more beneficial neuromodulation (radial error, mean radial error, suppressed midfrontal *θ* rhythm power). To ensure that positive SMD values consistently represented beneficial effects across all studies, the *d*-values calculated for all negative indicators underwent sign reversal (multiplied by −1). Heterogeneity was evaluated using the *χ^2^* test with a significance threshold of *α* = 0.1. Studies were deemed homogeneous when the *I^2^* statistic was <50%, and a fixed-effects model was applied to pool effect sizes. When *I^2^* ≥ 50% (indicating substantial heterogeneity), a random-effects model was applied. Potential sources of heterogeneity were investigated through subgroup analyses or sensitivity analyses. A two-tailed α = 0.05 was adopted to determine statistical significance for all analyses.

## 3 Results

### 3.1 Subsection

A total of 289 articles were retrieved from electronic databases according to the predefined search strategy: 32 from PubMed, 99 from Web of Science, 78 from Scopus, and 80 from EBSCO. No additional studies were identified from other sources. Following the removal of 152 duplicate records, 5 non-English language articles, and 25 studies excluded for other reasons (such as letters and conference abstracts), the titles and abstracts of the remaining 107 articles were systematically screened. Of these, 67 articles were excluded for the following reasons: no EEG and putting data, subjects not participating in putting training, or studies limited to systematic reviews or meta-analyses. Subsequently, full-text reviews were conducted for the remaining studies, with 9 non-randomized controlled trials, 8 studies failing to meet intervention criteria, and 11 studies with insufficient outcome indicators excluded. Finally, 12 eligible articles involving 424 subjects who underwent putt training with EEG measurements were included. The flowchart for the literature screening process is presented in Figure 1.

Twelve studies were included in this meta-analysis, involving 424 subjects, comprising 88 elite golfers, 40 mid-to-high handicap golfers (handicap ≥10), 56 Non-handicap golfers, and 240 novices. The EEG interventions targeted a range of frequency bands and brain regions. Protocols included enhancing SMR (12–15 Hz) at the central region (Cz) (Wu et al., 2024a; Afrash et al., 2023; Pourbehbahani et al., 2023; Cheng et al., 2015), modulating Mu rhythm (8–13 Hz) at Cz (Wang et al., 2023; Afrash et al., 2023; Wang et al., 2022), and suppressing FMT (4–7 Hz) or alpha (8–12 Hz) at Fz (Chen et al., 2022; Afrash et al., 2023; Ring et al., 2015). Other studies involved extensive putting practice without neurofeedback (Abdoli et al., 2025; Wang et al., 2024) or examined differences in neural coherence based on psychological traits (Zhu et al., 2011). The training regimens varied from single-session interventions (Chen et al., 2022; Wu et al., 2024a; Wang et al., 2023; Zhu et al., 2011; Wang et al., 2022) to multiple sessions conducted over several weeks (Afrash et al., 2023; Pourbehbahani et al., 2023; Cheng et al., 2015; Ring et al., 2015), with one longitudinal study tracking participants over a 3-month period (Wang et al., 2024). Putting tasks were conducted at distances ranging from 2 to 4 m, with trial counts varying from 40 to 300 putts per study. Pressure manipulations were implemented in several studies using methods such as cash rewards, performance rankings, and social evaluation (Wu et al., 2024a; Zhu et al., 2011; Ring et al., 2015). Primary outcomes encompassed both putting performance metrics, including radial error, putting success rate, and performance consistency, and EEG measures, such as absolute power in specific frequency bands and functional connectivity between brain regions. Data related to study characteristics are detailed in Table 1.

**Table 1:** Description of included studies.

Author	N(I/C)	Handicap (I/C)	Intervention type	Intervention duration	EEG neurophysiological Indicators	Putting outcome indicators
Abdoli et al. (2025)	15/15	Both novice	Putting Practice/No	3 days, 100 putts/d (10 × 10 reps/session)	F (F3, Fz, F4), C (C3, Cz, C4), P (P3, Pz, P4); Low *α* (8–10 Hz), High α (10–12 Hz); Abs power (log); 2000 ms pre/post lever–press	MRE (cm), SRE (cm), BE (cm)
Afrash et al. (2023)	32/32	Both novice	Cz–SMR NFT (12–15 Hz, enhance)/Fz–α NFT (8–12 Hz, suppress)/Cz–Mu NFT (8–13 Hz, suppress)/Sham	6 sess (3×/wk., 2w); 20 min NFT + 3 × 12 putts/sess	Cz (SMR/Mu), Fz (α); SMR (12–15 Hz), α (8–12 Hz), Mu (8–13 Hz); Abs power; rest (eyes open/closed), putt task	Radial error (cm)
Chen et al. (2022)	12/12	FSI:12.00 ± 11.02/TI:14.00 ± 7.38/SC:18.00 ± 8.86	Fz–FMT NFT (4–7 Hz, suppress: FSI/TI)/Sham	1 sess (1.5 h, 50 putts:10 × 5 reps)	Fz; FMT (4–7 Hz); Abs power; putt prep, rest	Success rate (40 putts)
Cheng et al. (2015)	8/8	0.00 ± 3.90	Cz–SMR NFT (12–15 Hz, enhance)/Sham	8 sess (2×/wk., 4wk); 30–45 min NFT/sess	Cz; SMR (12–15 Hz); Rel power; lever–press prep, rest	MRE (cm), SD (cm)
Gallicchio and Ring (2019)	16/16	Non-handicap	Randomized/blocked target practice	120 putts (20 baseline, 80 tests, 20 retention)	F (F3, Fz, F4), T (T7, F7, CP5), C (C3, Cz, C4), P (P3, Pz, P4), O (O1, Oz, O2); α (8–12 Hz); Abs/Rel power; lever–press prep	Radial error (cm), Len error (cm), Ang error (°)
Pourbehbahani et al. (2023)	20/20	Both novice	Cz–SMR NFT (12–15 Hz, enhance)/Pseudo–Feedback	6sess (1×/d, 6d); 20 min NFT + 3 × 12 putts/sess	Cz; SMR (12–15 Hz); Abs power; rest, push–button task	MRE (cm)
Ring et al. (2015)	12/12	23.00 ± 6.62/23.33 ± 4.62	Fz Zone 10–12 Hz High Alpha Suppression NFT (simultaneously suppressing 4–8 Hz theta)/Pseudo–Feedback (playback of paired NFT group feedback recordings).	3sess (1 h NFT/sess); 12 × 5 min putt blocks/sess	Fz; High α (10–12 Hz), θ (4–8 Hz); Rel power; lever–press prep	MRE (cm)
Wang et al. (2022)	10/10	Both novice	Cz–Mu NFT (8–13 Hz, enhance/suppress) vs. Sham	30–45 min (seated→standing, 70–80% success)	Cz (C3/C4); Mu (8–13 Hz) (+θ4–7 Hz, β14–20 Hz control); Abs power; joystick prep	MRE (cm); Subjective: Action control (11 pt), Stress (11 pt)
Wang et al. (2023)	10/10	Both novice	Cz–Mu NFT (8–13 Hz, enhance/suppress)/Sham	30–45 min (seated→standing, 70–80% success)	Cz (C3/C4); Mu (8–13 Hz) (+θ4–7 Hz, β14–20 Hz control); Abs power; joystick prep	MRE (cm); Subjective: Action control (11 pt), Stress (11 pt)
Wang et al. (2024)	41/41	Both novice	Repeated Practice/No practice	Longitudinal (7–22wk, ~12.4wk); ~25.9 sess	Fz–P3/P4 (frontoparietal), Cz–C3/C4 (sensorimotor); 8–13 Hz; Virtual inter–site PC; putt prep	Avg score (60 putts), SD
Wu et al. (2024a, 2024b)	44/44	0.44 ± 2.14	Cz/CPz–SMR NFT (12–15 Hz, enhance)/CON (no NFT) (cross–over)	30 min NFT + 60 putts	Cz/CPz; SMR (12–15 Hz), High α (10–12 Hz); Abs power, CSD; putt prep	Success rate (60 putts), SD
Zhu et al. (2011)	8/8	Both novice	High Vs. low conscious action control	10 putts tasks	T3–Fz, T4–Fz; α1 (8–10 Hz), α2 (10–12 Hz); Coherence (Fisher z); 4 s pre–putt	Avg distance (cm to target block)

I/C, Intervention group/Control group; NFT, Neurofeedback; SMR, Sensorimotor rhythm; FMT, Frontal midline theta; α, Alpha; θ, Theta; β, Beta; FSI, Function-specific instruction; TI, Traditional instruction; SC, Sham control; Abs, Absolute; Rel, Relative; PC, Phase coherence; CSD, Current source density; MRE, Mean radial error; SRE,subjective center of gravity radial error; BVE, Bivariate error; SD, Standard deviation; sess, Session; d, Day; wk, Week; pt, Point.

### 3.2 Quality assessment of the included literature

**Figure 2:**
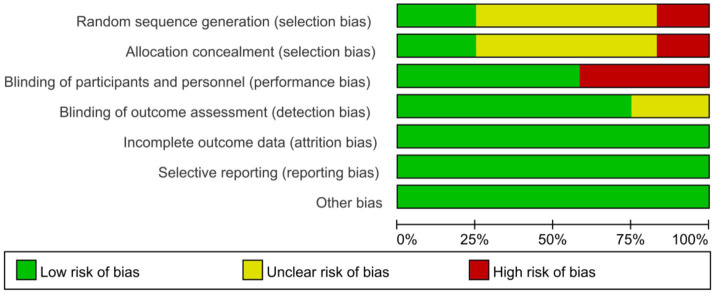
Risk of bias graph.

**Figure 3:**
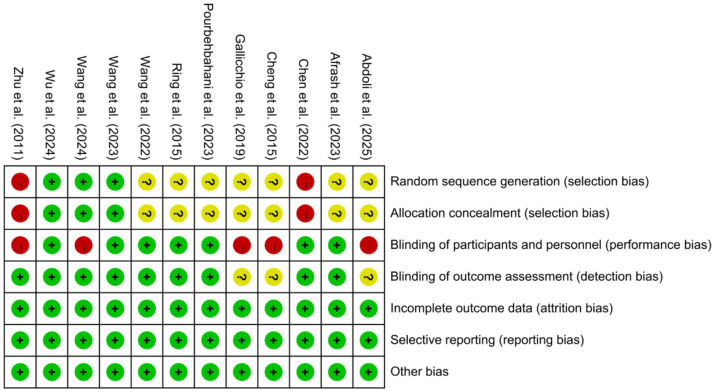
Risk of bias summary.

Based on the Cochrane Risk of Bias Assessment Scale, this study conducted a detailed assessment of the 12 selected studies, with the following results: for random sequence generation, 8 studies were at low risk, 2 at high risk, and 2 at unclear risk. In allocation concealment, 2 studies were rated as low risk, 2 as high risk, and 8 as unclear. Blinding of participants and personnel was challenging, with only 3 studies at low risk and most at high or unclear risk. For outcome assessment, 5 studies blinded assessors (low risk), while others either used objective measures or had unclear blinding (bias was unlikely due to objective outcomes). All 12 studies reported complete data (low risk for attrition bias) and fully reported prespecified outcomes (low risk for reporting bias), with no other bias identified. Overall, attrition, reporting, and other bias were low risk, but selection bias (unclear randomization/allocation) and performance bias (unblinded interventions) introduced uncertainty, as summarized in Figures 2, 3.

### 3.3 Meta-analysis

#### 3.3.1 Overall analysis

Since different indicators were used to evaluate putting performance (such as radial error, success rate, and absolute error) and EEG brain waves (such as alpha and theta waves) in the selected studies, SMDs were adopted as the combined effect size. For indicators with inconsistent directions, directional unification was performed before analysis to ensure all positive SMDs represented improved putting performance.

**Figure 4:**
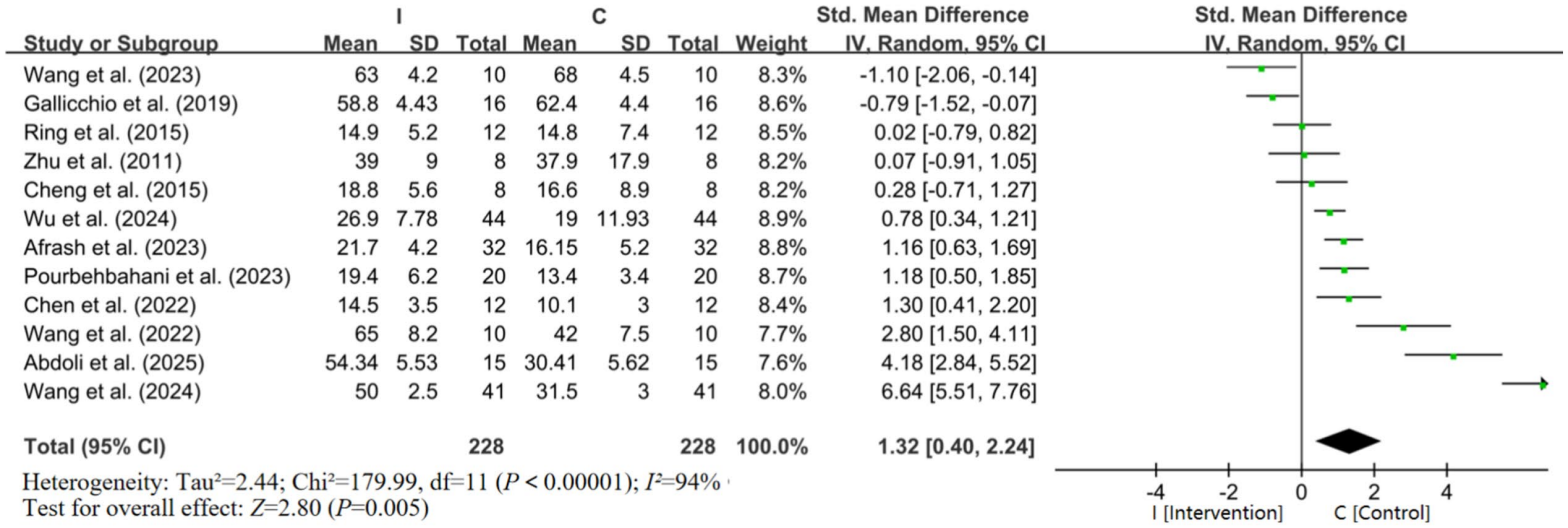
Forest plot of putting performance.

As shown in Figure 4, the pooled SMD for golf putting performance was 1.32 (95%CI, 0.40–2.24), with a statistically significant overall effect (*Z* = 2.80, *p* = 0.005). This indicates that, on average, the intervention had a significant positive impact on putting performance compared to control conditions. However, the substantial heterogeneity (*I^2^* = 94%) indicates that this overall estimate should be interpreted with caution, as it likely reflects variability in intervention effects across different study characteristics rather than a single homogeneous treatment effect. The high heterogeneity underscores the importance of exploring moderators through subgroup and meta-regression analyses. Sensitivity analyses were conducted as an exploratory (not prespecified) *post-hoc* procedure to identify sources of high heterogeneity. Five studies were excluded due to extreme effect size outliers, non-standard putting task distances, or unmatched active control conditions that distorted the global estimate (Wang et al., 2023; Wang et al., 2024; Sabate et al., 2012; Debnath et al., 2019; Pourbehbahani et al., 2023). After exclusion, heterogeneity dropped from 94 to 46%, and the pooled effect size remained significant (SMD = 0.76, 95%CI 0.40–1.12, *p* < 0.001), confirming robustness of the core finding (Table 2).

**Table 2:** Putting performance sensitivity analysis results.

Indicator	Included studies	Excluded studies	I/C (*n*)	*SMD* (95%CI)	*p*	*I^2^*	Heterogeneity of *p*-values
Putting performance	Chen et al. (2022), Wu et al. (2024a), Afrash et al. (2023), Abdoli et al. (2025), Gallicchio and Ring (2019), Zhu et al. (2011), Filho et al. (2021)	Wang et al. (2023), Wang et al. (2024), Sabate et al. (2012), Debnath et al. (2019), Pourbehbahani et al. (2023)	136/136	0.76 (0.40, 1.12)	<0.001	46%	0.09

Exploratory *post-hoc* sensitivity analysis excluding five outlier studies with non-standard protocols or extreme effect sizes.

#### 3.3.2 Subgroup analysis according to skill level

The subgroup difference tests revealed significant heterogeneity (*p* = 0.0003). Pairwise comparisons showed Elite vs. No-handicap (*p* < 0.001) and No-handicap vs. Novice (*p* = 0.002) differed significantly, while Mid-to-high handicap vs. No-handicap approached significance (*p* = 0.05). Notably, the analysis for players with no formal handicap showed a significant negative effect (*S*MD = −0.79, 95% CI: −1.52 to −0.07), which requires careful exploration in the Discussion section.

#### 3.3.3 Subgroup analysis according to intervention duration

Single-session subgroup: The pooled SMD was 1.69 (95% CI: 0.18–3.20), accounting for 66.0% of the total weight. This indicates a large positive effect, suggesting single-session interventions could effectively improve putting performance. Multiple-session subgroup: The SMD was 0.71 (95% CI: 0.10–1.31) with 34.0% weight, reflecting a moderate positive effect (Table 3). The test for subgroup differences was not significant (*p* = 0.23), indicating that intervention duration did not show a statistically detectable impact on effect size heterogeneity.

**Table 3:** Results from the subgroup meta-analyses.

Subgroup analysis	Classification	Included studies	*SMD* (95%CI)	Weight/%	Subgroup differences
Skill level	Elite	Wu et al. (2024a), Gallicchio and Ring (2019)	0.89 (0.47, 1.31)	17.3%	Elite vs. No-handicap (*p* <0.001), Mid-to-high handicap vs. No-handicap (*p* = 0.05), No-handicap vs. Novice (*p* = 0.002), Test for subgroup differences (*p* = 0.0003)
	Mid-to-high handicap	Chen et al. (2022), Zhu et al. (2011)	0.64 (−0.62, 1.90)	16.9%	
	No-handicap	Van Noordt et al. (2016)	−0.79 (−1.52, −0.07)	8.6%	
	Novice	Wang et al. (2023), Afrash et al. (2023), Abdoli et al. (2025), Wang et al. (2024), Sabate et al. (2012), Debnath et al. (2019), Filho et al. (2021)	2.10 (0.37, 3.84)	57.2%	
Intervention duration	Single-session	Chen et al. (2022), Wu et al. (2024a), Wang et al. (2023), Wang et al. (2024), Sabate et al. (2012), Debnath et al. (2019), Filho et al. (2021), Van Noordt et al. (2016)	1.69 (0.18, 3.20)	66.0%	The test was not significant (*p* = 0.23)
	Multiple-session	Afrash et al. (2023), Abdoli et al. (2025), Gallicchio and Ring (2019), Zhu et al. (2011)	0.71 (0.10, 1.31)	34.0%	
Intervention type	SMR-NFT	Wu et al. (2024a), Afrash et al. (2023), Abdoli et al. (2025), Gallicchio and Ring (2019)	0.88 (0.57, 1.19)	53.9%	The test was not significant (*p* = 0.27)
	Mu-NFT	Wang et al. (2023), Wang et al. (2024)	0.26 (−0.51, 1.03)	8.8%	
	FMT-NFT	Chen et al. (2022)	−1.92 (−2.92, −0.92)	6.5%	
	Non-EEG intervention	Zhu et al. (2011), Sabate et al. (2012), Debnath et al. (2019), Filho et al. (2021), Van Noordt et al. (2016)	1.05 (0.63, 1.46)	30.8%	

#### 3.3.4 Subgroup analysis according to intervention type

The type of intervention was not a statistically significant moderator of the overall effect (test for subgroup differences: *p* = 0.27). Among the EEG neurofeedback protocols, SMR-NFT showed a consistent, large, and significant effect on performance (SMD = 0.88, 95% CI: 0.57–1.19). FMT-NFT also demonstrated a large and significant effect (SMD = -1.92, 95% CI: −2.92 to −0.92), while Mu-NFT did not yield a significant improvement (SMD = 0.26, 95% CI: −0.51 to 1.03). Furthermore, non-EEG interventions were highly effective, producing a large and significant pooled effect size (SMD = 1.05, 95% CI: 0.63–1.46).

#### 3.3.5 EEG characteristics of different intervention types

**Figure 5:**
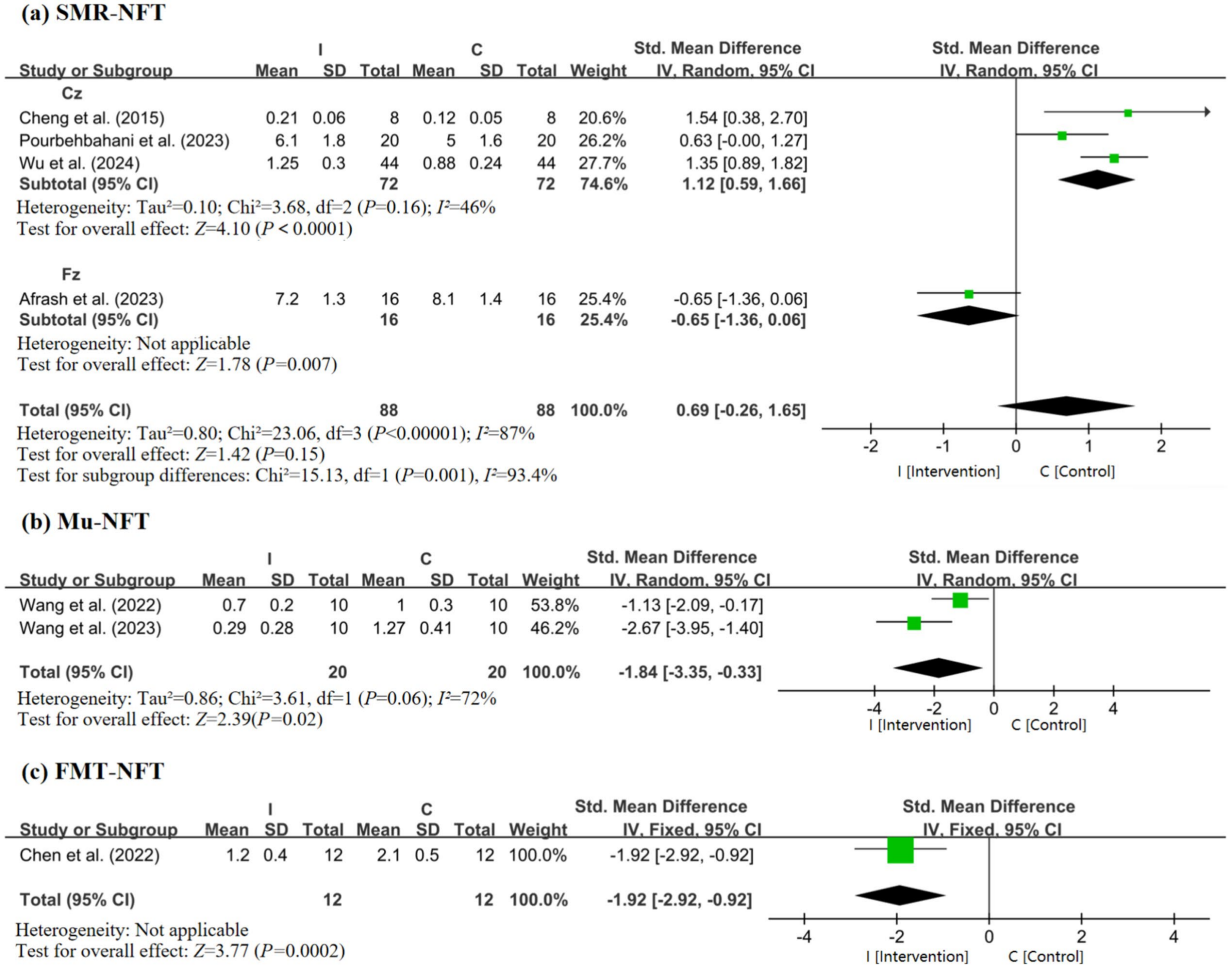
Forest plot of EEG intervention **(a)** SMR-NFT, **(b)** Mu-NFT, **(c)** FMT-NFT.

**Figure 6:**
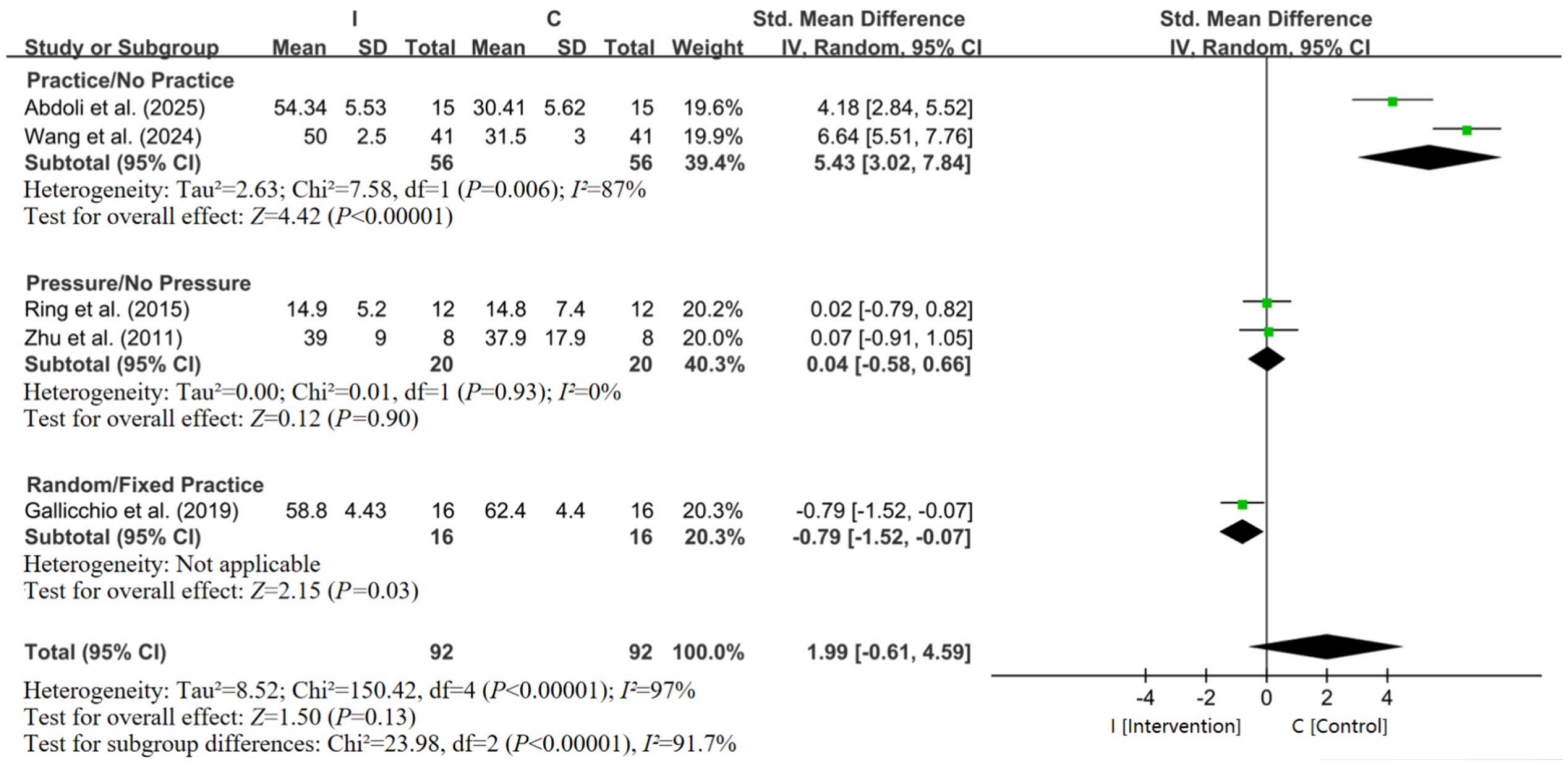
Forest plot of non-EEG intervention.

To further elucidate the efficacy of EEG-based neurofeedback (NFT) interventions, subgroup meta-analyses were conducted for three major modalities: SMR-NFT, Mu-NFT, and FMT-NFT. Three studies involving 72 participants were included (Wu et al., 2024a; Abdoli et al., 2025; Gallicchio and Ring, 2019). The pooled SMD was 1.12 (95% CI: 0.59–1.66; *Z* = 4.10, *p* < 0.0001) with low heterogeneity (*I^2^* = 46%), indicating a moderate-to-large positive effect of the Cz-site SMR-NFT on putting performance (Figure 5a). Fz-site SMR-NFT: a single study with 16 participants showed an SMD of −0.65 (95% CI: −1.36 to 0.06; *Z* = 1.78, *p* = 0.07), suggesting no significant effect. Mu-NFT intervention: Inhibiting Mu rhythm enhanced putting performance (Afrash et al., 2023). The pooled SMD was −1.84 (95% CI: −3.35 to −0.33; *Z* = 2.39, *p* = 0.02) with moderate heterogeneity (*I^2^* = 72%), indicating a large negative effect (Figure 5b). As shown in Figure 5c, the SMD was −1.92 (95% CI: −2.92 to −0.92; *Z* = 3.77, *p* = 0.0002), reflecting a large negative effect of FMT-NFT on putting performance. To investigate the effects of non-EEG interventions on golf putting performance, the subgroup meta-analyses were conducted stratified by electrode recording sites: Practice/No Practice, Pressure/No Pressure, and Random/Fixed Practice Combining all non-EEG intervention studies, the pooled SMD was 1.99 (95% CI: −0.64 to 4.59; *Z* = 1.50, *p* = 0.13) with high heterogeneity (*I^2^* = 97%). The test for subgroup differences was statistically significant (*p* <0.00001). Overall, non-EEG interventions suggest that deliberate practice improves putting performance. Subgroup analysis for the non-EEG intervention is shown in Figure 6.

## 4 Discussion

This meta-analysis synthesized findings from 12 studies to elucidate the effects of interventions on golf putting performance, while acknowledging substantial heterogeneity (*I^2^* = 94%) that necessitates nuanced interpretation of subgroup and EEG-specific outcomes. In line with standard meta-analytic practice, the overall pooled effect is presented only as a global descriptive summary; all the mechanistic and practical conclusions are exclusively based on prespecified subgroup analyses that address the substantial heterogeneity. Beyond the skill levels and intervention protocols that have been examined, variations in methodological and contextual factors contribute to the wide distribution of effect sizes. Inconsistencies in task parameters represent a significant potential source of variation, as studies employed widely differing putt distances (2–4 m) and trial counts (40–300 trials). Distance directly influences task difficulty, while trial count affects learning- curve saturation and measurement stability, both factors that modulate the manifestation of intervention effects. The diversity of stress manipulation methods may introduce distinct types of psychological load. Although stress is a core element in competitive golf, the heterogeneity of its induction methods may influence anxiety, motivation, and neural activity, thereby non-systematically modulating the effects of neurofeedback interventions. Although subgroup differences in intervention duration (single session vs. multiple sessions) did not reach statistical significance, subtle variations in neurofeedback dosage (total training time, single session duration, and training frequency) may contribute to residual heterogeneity. The diversity of control group types (such as sham feedback, no training, low-consciousness motor control, and other behavioral interventions) implies that intervention effects were relative to different baselines. The less similar the control group activity was to the intervention, the greater the potential for overestimating the intervention’s specific effect; in contrast, comparisons against other active interventions (e.g., behavioral training) highlight relative advantages. This variation in comparators was a common source of heterogeneity in meta-analyses. The effect sizes observed in this study should be interpreted cautiously as composite estimates of effects under specific methodological and contextual conditions, rather than as a universal value. Future research should focus on reducing these methodological variations through standardized task paradigms, control group designs, and dose reporting. Subgroup analyses were conducted according to participants’ skill levels, intervention duration, and intervention types, while a meta-analysis examined EEG characteristics in specific brain regions for each intervention type.

Novice golfers exhibited a positive effect of interventions (SMD = 2.10), as they possess less consolidated motor representations and are more responsive to interventions that shape sensorimotor integration (Edwards et al., 2019). Novices, with their less consolidated motor engrams, appear to possess a wider window for neurobehavioral adaptation, allowing interventions to readily facilitate the formation of efficient cortical networks (Abdoli et al., 2025). SMR-NFT may be more effective in novices compared to experienced golfers with more rigid movement schemas. Gallicchio and Ring (2019) analyzed alpha power changes in the frontal lobe region of interest (ROI) during the motor preparation phase to investigate the impact of cognitive inhibition on putting performance. Moreover, considering that the intervention group received random target training, the study revealed that, in contrast to fixed target training, random target training had an adverse impact on the putting performance of no-handicap golfers. The negative effect observed in the no-handicap subgroup (SMD = −0.79) is based on one study and requires replication before firm conclusions can be drawn. Elite golfers showed a moderate positive effect (SMD = 0.89), likely because interventions refined already efficient cortical processes and optimized pre-movement SMR oscillations to reduce cognitive interference (Wu et al., 2024a; Rees et al., 2007; Beattie et al., 2011). While the overall test for subgroup differences by intervention duration was not significant, the pattern of results is instructive for practical implementation. The large but highly variable effect of single-session interventions suggests a role in rapid state regulation, potentially ideal for pre-competition priming in athletes who already possess an established skill (Wu et al., 2024b; Pereira et al., 2025; Tosti et al., 2024). In contrast, the smaller but more consistent effect of multiple sessions aligns with inducing longer-term trait changes in neural circuitry, which is more appropriate for foundational skill acquisition in novices (Wang et al., 2022; Williams and Hodges, 2023; Kraeutner et al., 2024). SMR power emerges as a key mechanism for optimizing golf putting. Meta-analytic findings showed that Cz-region SMR enhancement (via NFT) correlated with improved putting accuracy (reduced MRE) and stability (lower SD) (Abdoli et al., 2025; Wang et al., 2024). This supports theories of neural efficiency: SMR oscillations in central sensorimotor regions reflect inhibitory control over non-task-related neural activity, promoting movement automation (Pourbehbahani et al., 2023; Mohamed et al., 2025). Babiloni et al. reported event-related desynchronization of SMR prior to successful putts in high-resolution EEG, which coincided with a 38% reduction in distance control error in elite vs. novice golfers during a 3-meter task, a mechanism potentially linked to SMR-mediated inhibition of spinal motor neuron noise (Pichiorri et al., 2011). In contrast, modulation of other rhythms yielded less consistent or even negative effects. Mu rhythm (8–13 Hz) inhibition showed a negative effect (SMD = −1.84) in two studies, possibly because Mu oscillations underlie action observation and mirror neuron function (Wang et al., 2023; Wang et al., 2022; LaMarca et al., 2023; Datko et al., 2018); excessive inhibition may disrupt internal models of putting kinematics. Similarly, FMT (4–7 Hz) suppression appears to be associated with impaired putting performance (SMD = −1.92), likely reflecting impaired cognitive control. FMT is implicated in working memory and attentional allocation (Ahn et al., 2025), and over-suppression of this rhythm may disrupt cognitive control processes relevant to putting.

These protocols, often theorized to reduce interference, might instead be removing essential neural components for optimal performance, revealing a critical boundary condition for inhibitory neurofeedback (Chen et al., 2022; Shtossel et al., 2023). For novices, Cz-SMR enhancement may be employed for neural construction; for elite athletes, training may focus on maintaining SMR stability under stress; non-specific FMT suppression should be avoided for all groups.

The sensitivity analysis revealed that the high heterogeneity in the overall meta-analysis was largely driven by a subset of five studies. Notably, after excluding these studies, heterogeneity was substantially reduced (*I^2^* = 46%) and the pooled effect remained significant (*SMD* = 0.76). These findings suggest that while methodological variability across studies contributes to heterogeneity, the positive direction of effect is relatively robust. The sensitivity analysis was exploratory, and the results should be interpreted with appropriate caution. The five excluded studies included both the EEG-NFT and non-EEG training interventions, indicating that heterogeneity stems from multiple sources rather than a single study type. Regarding intervention type, EEG neurofeedback, distinguished by its precise targeting of brain region–rhythm–behavior mechanisms, and non-EEG behavioral interventions each exert positive effects, but EEG-NFT offers superior mechanistic specificity tailored to golf putting’s sensorimotor demands. Previous studies have demonstrated that the Cz region of SMR-NFT has a positive correlation with putter performance. For novices, this modulation yielded persistent benefits: Cz-SMR power increases correlated with stable MRE reductions even at follow-up, indicating the establishment of robust motor control patterns (Lu et al., 2025; Wang et al., 2020; Christie et al., 2020). For elite golfers, the Cz-SMR elevation aligned with their need for “low cognitive interference,” coinciding with higher putting success rates, increased subjective relaxation, and reduced conscious movement control–directly meeting the neural demands of elite-level automated performance (Ahn et al., 2025; Exel and Dabnichki, 2024). The Cz region of Mu rhythm inhibition targeted a complementary mechanism, correlating with reduced MRE and heightened subjective action control (Wang et al., 2023; Wang et al., 2022). The novices, who require greater sensorimotor engagement, benefited from Mu inhibition but struggled to enhance the Cz-Mu power through function-specific instruction (FSI), highlighting that Mu modulation efficacy is skill level dependent (complex tasks demand Mu suppression, not enhancement, for novices). Fz region interventions (Alpha-wave inhibition and FMT modulation) lacked consistent efficacy. For instance, the “Alpha group” targeted Fz-region 8–12 Hz alpha inhibition, but did not replicate the benefits of the Cz-region modulation, likely because the Fz was less aligned with putting’s sensorimotor core than the Cz (Wang et al., 2020; Abdoli et al., 2025; Wang et al., 2024; Chueh et al., 2023). Similarly, FMT modulation at Fz, designed to reduce conscious effort, failed to match with the Cz-SMR’s automation–promoting effects, underscoring that EEG-NFT efficacy hinges on aligning target regions/rhythms with the task’s neural substrates (Chen et al., 2022; Yu et al., 2025; Cheng et al., 2024). Non-EEG interventions also produced large positive effects (*SMD* = 1.05), but relied on broader experience-dependent plasticity (Abdoli et al., 2025; Ring et al., 2015; Van de Vel et al., 2013), such as error-based adaptation or task variation (random target presentation to refine visual spatial integration). These interventions shape motor learning through behavioral repetition but lack the precision to target specific neural mechanisms, making them less tailored to individual skill level neural needs (Wu et al., 2014; Maier et al., 2019; Levin and Demers, 2021). Non-EEG interventions leverage general motor learning principles, while EEG-NFT, particularly when targeting the Cz-region SMR or Mu rhythms, delivers “mechanism-specific” optimization by aligning neural modulation with putting’s sensorimotor demands and skill-level-dependent neural profiles (Smith et al., 2013; Rauter et al., 2015). Regarding intervention type, EEG-NFT, defined by its precise targeting of brain-region–rhythm–behavior mechanisms, and non-EEG behavioral interventions both yield large positive effects on golf putting. However, EEG-NFT provides superior mechanistic specificity tailored to the task’s sensorimotor demands and skill-level-dependent neural needs (Chen et al., 2022; Pourbehbahani et al., 2023; Corrado et al., 2024). Future personalized training may require concurrent monitoring of psychological states (via brief questionnaires or autonomic indicators). Training protocols should dynamically adjust, prioritizing validated SMR-enhancing protocols proven to promote relaxation and automation when high-anxiety states are detected, rather than directly challenging high-load cognitive control regions.

## 5 Conclusion

This meta-analysis provides preliminary evidence that golf putting performance may be enhanced through interventions aligned with the neurobehavioral demands of the skill and individual expertise levels, though findings should be interpreted cautiously given the limited evidence base and substantial heterogeneity across studies. Both the EEG-based neurofeedback protocols and other training interventions appear to influence putting performance by targeting neural regulation, motor learning, and cognitive control mechanisms, with SMR-NFT at Cz emerging as the most promising protocol for promoting movement automation through sensorimotor integration. Skill level appears to moderate intervention efficacy, with novices demonstrating neuroplastic potential for substantial improvement, while elite performers may benefit from refined neural efficiency. However, the observed negative effects for inhibitory protocols (Mu-NFT and FMT-NFT), derived from a small number of studies, suggest potential boundary conditions where suppressing relevant neural oscillations may disrupt performance-critical processes. These findings, while identifying promising directions for tailored interventions integrating EEG-NFT and non-EEG approaches, remain preliminary and warrant cautious interpretation pending further replication with larger and more homogeneous samples.

## Data Availability

The original contributions presented in the study are included in the article/Supplementary material, further inquiries can be directed to the corresponding author.
