# Supplementary material for: Effects of EEG neurofeedback and training interventions on golf putting performance: a systematic review and meta-analysis
Source: Front Psychol. 2026 Apr 23;17:1736851. doi: 10.3389/fpsyg.2026.1736851 (PMC13149423; doi:10.3389/fpsyg.2026.1736851)
Supplement: Supplementary file 1 [file Supplementary_File_1.docx]

**Supplementary file 1**

**1.Databases：**PubMed, Web of Science, Scopus, and EBSCO.

**2.Search terms（Subject terms and keywords）**

Searches were conducted using Medical Subject Headings (MeSH) and keywords associated with the PICO elements.

**Population**

**MeSH Terms:** Golf, Golfer

**Keywords:** Golf putting, Golfer, Putting performance, Amateur golfer, Professional golfer, Elite golfer, Low handicap golfer, High handicap golfer, Novice, Golf putting accuracy, Golf putting success

**Intervention**

**MeSH Terms:** Electroencephalography (EEG), Neurofeedback (NFT)

**Keywords:** EEG, Electroencephalographic, Brain wave, Alpha wave, Beta wave, Theta wave, Neural oscillation, Visual fixation, Neurofeedback training, EEG biofeedback

**Comparison**

**MeSH Terms:** Control Groups, Comparative Study, Skill Level

**Keywords:** Control, Comparison, Expert vs novice, Low handicap vs high handicap, Intervention vs control, Stress vs non-stress condition,Training vs no Training

**Outcome**

**MeSH Terms:** Golf Putting, Motor Performance, Performance Measurement

**Keywords:** Putting success rate, Putting accuracy, Distance control, Motor consistency, EEG power, Sensorimotor rhythm, Stress resilience, Neurobehavioral profile

**3.Search strings**

**PubMed:** (golf [Title]) AND ("golf putting" OR "golf putt"OR "golf putting performance" OR "golf putting accuracy")AND( "Electroencephalography" OR "EEG" OR "electroencephalographic" OR "brain wave*" OR "alpha wave*" OR "beta wave*" OR "theta wave*" OR "neural oscillation*" OR "neurofeedback" OR "EEG feedback")

**Web of Science:** (golf [TI]) AND (swing or biomechanics* OR

kinematic* OR kinetic* OR dynamic* OR angle OR velocity* OR speed OR torque OR moment OR force OR GRF OR mechanic* OR power OR work OR energy* [TS]);

(TS=(golf)) AND (TS=(golf putting OR golf putt OR golf putting performance OR golf putting accuracy OR golf putting success )) AND (TS=(Electroencephalography OR EEG OR brain wave OR alpha wave OR beta wave OR theta wave OR neural oscillation OR neurofeedback OR EEG feedback))

**Soups:**( ( TITLE-ABS-KEY ( golf ) OR TITLE-ABS-KEY ( golf AND putting ) OR TITLE-ABS-KEY ( golf AND putt ) OR TITLE-ABS-KEY ( golf AND putting AND performance ) OR TITLE-ABS-KEY ( golf AND putting AND accuracy ) OR TITLE-ABS-KEY ( golf AND putting AND success ) ) ) AND ( (TITLE-ABS-KEY ( electroencephalography ) OR TITLE-ABS-KEY ( eeg ) OR TITLE-ABS-KEY ( brain AND wave ) OR TITLE-ABS-KEY ( alpha AND wave ) OR TITLE-ABS-KEY ( beta AND wave ) OR TITLE-ABS-KEY ( theta AND wave ) OR TITLE-ABS-KEY ( neural AND oscillation ) OR TITLE-ABS-KEY ( neurofeedback ) OR TITLE-ABS-KEY ( eeg AND feedback ) ) )**EBSOC:**golf OR ( golf putting OR golf putt OR golf putting performance OR golf putting accuracy OR golf putting success ) AND Quiet Eye OR QE OR quiet eye training OR quiet eye duration OR quiet eye measurement OR quiet eye fixation OR Electroencephalography OR EEG OR brain wave OR alpha wave OR beta wave OR theta wave OR neural oscillation OR neurofeedback OR EEG feedback

**EBSOC:**golf OR (golf putting OR golf putt OR golf putting performance OR golf putting accuracy OR golf putting success) AND (Electroencephalography OR EEG OR brain wave OR alpha wave OR beta wave OR theta wave OR neural oscillation OR neurofeedback OR EEG feedback)
